# Telomerase deficiency and dysfunctional telomeres in the lung tumor microenvironment impair tumor progression in NSCLC mouse models and patient-derived xenografts

**DOI:** 10.1038/s41418-023-01149-6

**Published:** 2023-04-21

**Authors:** Sergio Piñeiro-Hermida, Giuseppe Bosso, Raúl Sánchez-Vázquez, Paula Martínez, Maria A. Blasco

**Affiliations:** Telomeres and Telomerase Group, Molecular Oncology Program, Spanish National Cancer Centre (CNIO), Melchor Fernández Almagro 3, Madrid, E-28029 Spain

**Keywords:** Non-small-cell lung cancer, Cancer models

## Abstract

Non-small cell lung cancer (NSCLC) is a leading cause of cancer death. Tumor progression depends on interactions of cancer cells with the tumor microenvironment. Here, we find increased copy number and mRNA expression of the catalytic subunit of telomerase, *TERT*, in tumors from NSCLC patients, contributing to a lower survival. Moreover, *TERT* expression in NSCLC patients from the TCGA cohort is mainly associated to the reduced infiltration of CD8^+^ T lymphocytes, as well as to increased infiltration of myeloid-derived suppressor cells (MDSCs). We also show that TERT deficiency and dysfunctional telomeres induced by 6-thio-dG treatment in mice reduced lung tumor implantation and vascularization, increased DNA damage response, cell cycle arrest and apoptosis, as well as reduced proliferation, inflammation, lung tumor immunosupression and invasion upon induction of a Lewis lung carcinoma (LLC). Furthermore, 6-thio-dG-treated human NSCLC xenografts exhibited increased telomere damage, cell cycle arrest and apoptosis, as well as reduced proliferation, resulting in a reduced tumor growth. Our results show that targeting telomeres might be an effective therapeutic strategy in NSCLC.

## Introduction

Lung cancer is a major public health problem worldwide, being the leading cause of cancer death with a five-year survival rate of 21% due to the long-term ineffectiveness of current therapies and late stage at time of diagnosis. Specifically, the predominant lung cancer subtype, the non-small-cell lung cancer (NSCLC), accounts for 85% of lung cancer-associated deaths [1, 2].

Tumors are composed of tumor cells as well as resident and infiltrating host cells, secreted factors and extracellular matrix proteins, collectively known as the tumor microenvironment. Tissue-resident and peripherally recruited immune cells, fibroblasts and endothelial cells are considered key elements within the tumor microenvironment. Notably, tumor progression is profoundly influenced by interactions of cancer cells with the tumor microenvironment, which can also shape therapeutic responses and resistance. Thus, the tumor microenvironment is key for the development and implementation of new drugs aimed at blocking cancer progression [3, 4].

Telomeres are heterochromatic structures at the ends of eukaryotic chromosomes, which are essential for chromosome stability. In mammals, telomeric DNA consists of TTAGGG tandem repeats bound by a 6-protein complex known as shelterin [5, 6]. With each cell division, telomeres shorten due to the incomplete replication of chromosome ends [7, 8]. Telomere shortening can be compensated through the de novo addition of telomeric repeats by telomerase, a reverse transcriptase composed of a catalytic subunit (TERT) and an RNA component (Terc), used as a template for telomere elongation [9].

TERT is downregulated in the majority of tissues after birth [10, 11]. In contrast, TERT is reactivated in 90% of human tumors to maintain a minimum functional telomere length to allow cancer cell division [12]. Similarly, TERT is over-expressed in mouse tumors [13]. Of interest, TERT is suggested to regulate key components of the tumor microenvironment such as inflammation and immunosuppression, and fibroblast activation in multiple cancers other than NSCLC [12]. Thus, telomerase is considered an anti-cancer target [14, 15].

Here we address the effects of telomerase deficiency in the lung tumor microenvironment, as well as the anti-tumor activity of 6-thio-2´-deoxyguanosine (6-thio-dG). 6-thio-dG is a nucleoside analog that is incorporated into telomeric DNA by telomerase expressing cells, without inhibiting telomerase activity, leading to telomere dysfunction, genomic instability and cell death [16].

## Results

### Increased amplification frequency, copy number values and mRNA expression of TERT in NSCLC patients, and reduced tumor implantation in TERT-deficient mice upon lung tumor induction

**Fig. 1:**
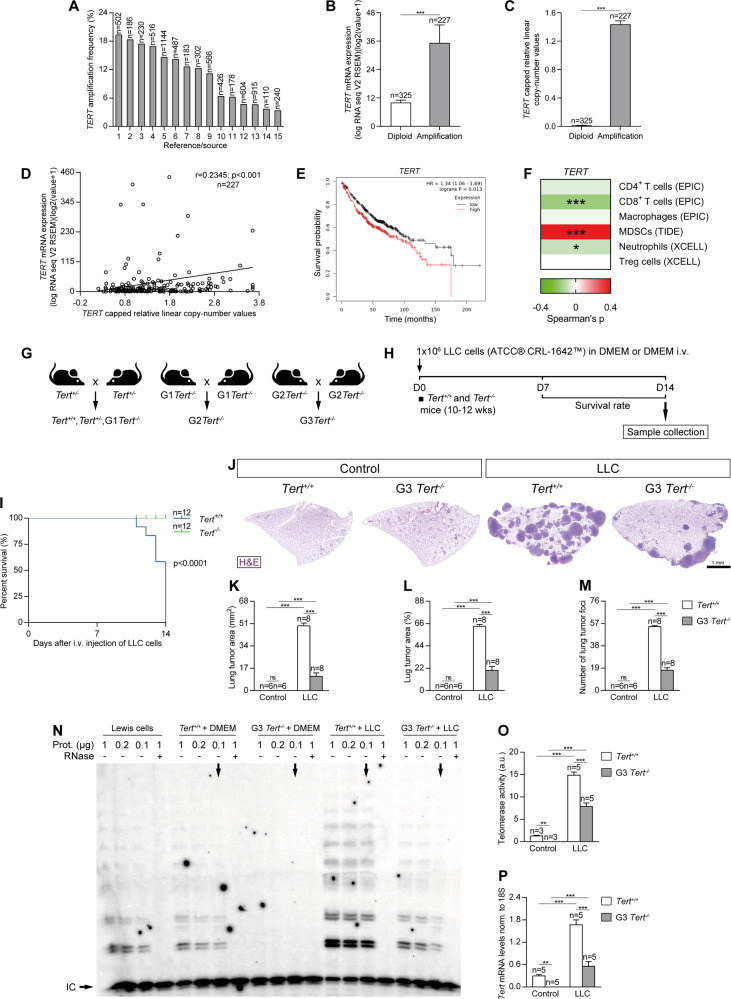
Increased amplification frequency, copy number values and mRNA expression of TERT in NSCLC patients, and reduced tumor implantation in TERT-deficient mice upon lung tumor induction. Amplification frequency (**A**), copy number values (**B**) mRNA expression levels of *TERT* (**C**) and Pearson correlation of mRNA expression with copy number values of *TERT* (**D**) in lung tissues from NSCLC patients, and survival probability in NSCLC patients with high and low TERT expression (**E**) obtained from the Kaplan–Meier Plotter database. **F** Correlation between the expression of *TERT* and immune infiltrates in NSCLC patients from the TCGA using the TIMER 2.0 database. **G**, **H** Generation of *Tert*^*+/+*^ and G3 *Tert*^*−/−*^ mice and protocol for the induction of Lewis Lung Carcinoma (LLC). **G** Heterozygous *Tert*^*+/−*^ mice were crossed to obtain *Tert*^*+/+*^ and G1 *Tert*^*−/−*^ mice, and successive crosses between G1 *Tert*^*−/−*^ and then G2 *Tert*^*−/−*^ were set to generate G3 *Tert*^*−/−*^ mice. **H** 1 ×10^6^ Lewis cells suspended in 100 µl of DMEM or equal volume of DMEM (controls) were injected via the tail vein of 10–12 weeks old *Tert*^*+/+*^ and G3 *Tert*^*−/−*^ male mice on day 0 (D0). An in vivo follow follow-up of survival was carried out until sample collection on day 14 (D14). Kaplan–Meier survival curves (**I**), representative images of LLC-challenged *Tert*^*+/+*^ and G3 *Tert*^*−/−*^ lungs and controls (H&E) (**J**), and quantification of lung tumor area (**K**, **L**) and foci (**M**) in *Tert*^*+/+*^ and G3 *Tert*^*−/−*^ mice. **N** Representative Telomeric repeat amplification protocol (TRAP) using S-100 lung extracts from LLC-challenged *Tert*^*+/+*^ and G3 *Tert*^*−/−*^ mice and controls, where protein concentration is indicated. Extracts were treated (+) or not (−) with RNase as a negative control (exposition time: 48 h). An internal control (IC) for PCR efficiency was also included and arrows indicate the lane used for quantification. **O** Quantification of Telomerase activity in lung extracts from LLC-challenged *Tert*^*+/+*^ and G3 *Tert*^*−/−*^ mice and controls expressed in arbitrary units (a.u). **P** Lung tissue mRNA expression levels of *Tert* normalized to 18S expression in *Tert*^*+/+*^ and G3 *Tert*^*−/−*^ mice. Data are expressed as mean ± SEM (the number of mice is indicated in each case). ***p < 0.001 (Dunn–Sidak test for multiple comparisons and Mann–Whitney or unpaired *t* tests to compare 2 independent groups). Survival was assessed by the Kaplan–Meier analysis, using the log Rank (Mantel–Cox) test).

As TERT is commonly over-expressed and mutated in multiple human cancers [17], here we first explored genomic alterations in the *TERT* locus and their correlation with survival in non-small cell lung cancer (NSCLC) patients (Fig. 1A–E). To this end, we analyzed data from different studies included in the cBio Cancer Genomics Portal (cBioPortal). On average, we found an 11.02 % increased amplification frequency for *TERT* (Fig. 1A and Supplementary Table S1). *TERT* copy number values and mRNA expression levels were also significantly increased in tissue samples from NSCLC patients compared to diploid tissue (Fig. 1B, C). In addition, *TERT* mRNA expression was increased in 20 tumor types including lung adenocarcinomas (LUAD) and lung squamous cell carcinomas (LUSC) (data from The Cancer Genome Atlas (TCGA)) (Supplementary Fig. S1). Moreover, the increase in *TERT* mRNA expression was correlated with copy number values (Pearson correlation coefficient, *r* = 0.2345, *p* < 0.001) (Fig. 1D). Interestingly, NSCLC patients with high expression of *TERT* exhibited significantly worse survival rates after 2, 5 and 16 years compared with patients with low *TERT* expression (Fig. 1E and Suplementary Fig. S2). In addition, we have found a correlation between *TERT* expression and immune infiltrates in NSCLC patients from the TCGA using the TIMER 2.0 database (Fig. 1F). High TERT expression correlates with lower infiltration of CD8^+^ T cells (Spearman’s correlation = −0.177) and to a lower extend of neutrophils (Spearman’s correlation = −0.094). We also found that TERT expression in these patients is associated with increased infiltration of myeloid derived-suppressor cells (MDSCs) (Spearman’s correlation = 0.409), without affecting the infiltration of CD4^+^ T cells (Spearman’s correlation = −0.049), macrophages (Spearman’s correlation = −0.025) and treg cells (Spearman’s correlation = 0) (Fig. 1F).

Next, we assessed the role of TERT deficiency on lung tumor progression and in key components of the lung tumor microenvironment by using a NSCLC mouse model. For that purpose, wild-type mice (*Tert*^*+/+*^) and third generation (G3) telomerase-deficient mice (G3 *Tert*^*−/−*^) with short telomeres (C57BL/6 background) [18] were intravenously injected with Lewis Lung Carcinoma (LLC) cells (LL/2 (LLC1) (ATCC® CRL-1642™)) to generate lung tumors (Fig. 1G–H). Mice were sacrificed at 14 days post-injection and samples taken for further analysis. Of note, following induction of LLC, 100% of the G3 *Tert*^*−/−*^ mice survived compared to 100% mortality in the case of the *Tert*^*+/+*^ controls (Fig. 1I). In agreement with this, G3 *Tert*^*−/−*^ mice showed reduced tumor growth compared to *Tert*^*+/+*^ mice as indicated by a decreased lung tumor area and less tumor foci (Fig. 1J–M). We assessed telomerase activity by performing a telomeric repeat amplification protocol (TRAP) assay, and *Tert* mRNA expression levels in the lungs of our mouse cohorts by qPCR (Fig. 1N–P). As expected, telomerase activity was not detected in G3 *Tert*^*−/−*^ compared to *Tert*^*+/+*^ control mice. We found a significantly increased telomerase activity upon induction of LLC, being this increase higher in *Tert*^*+/+*^ compared to G3 *Tert*^*−/−*^ mice (Fig. 1N, O). Of note, Lewis cell extracts exhibited a lower telomerase activity compared to that observed in LLC-challenged mice (Fig. 1N), indicating a possible interaction of tumor cells with the microenvironment, that induces the expression of TERT in the tumor cells. Accordingly, total lung *Tert* mRNA expression upon LLC challenge was significantly higher in *Tert*^*+/+*^ mice compared to G3 *Tert*^*−/−*^ mice (Fig. 1P).

### Telomerase deficiency in the tumor microenvironment reduces lung tumor progression, invasion and vascularization reducing cell proliferation and increasing DNA damage response, cell cycle arrest and apoptosis

**Fig. 2:**
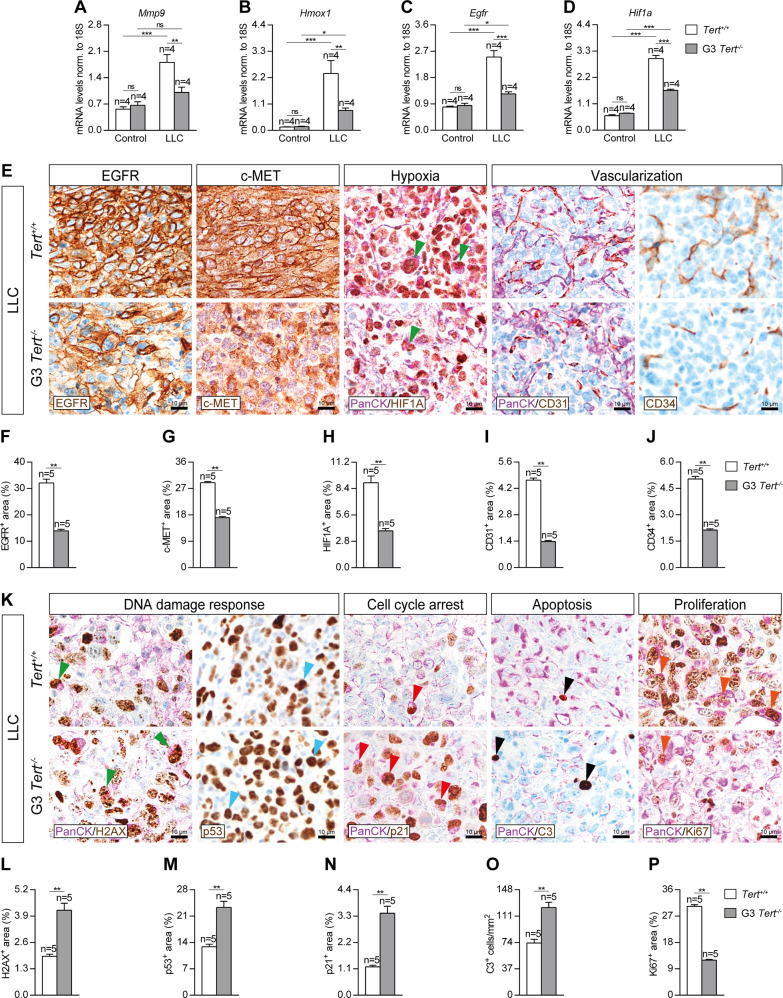
Telomerase deficiency in the tumor microenvironment reduces lung tumor progression, invasion and vascularization reducing cell proliferation and increasing DNA damage response, cell cycle arrest and apoptosis. Lung tissue mRNA expression levels of *Mmp9* (**A**), *Hmox1* (**B**), *Egfr* (**C**) (tumor progression markers) and *Hif1a* (hypoxia and tumor invasion) (**D**) normalized to 18S expression in *Tert*^*+/+*^ and G3 *Tert*^*−/−*^ mice. Representative lung immunostainings for EGFR (brown), c-MET (brown), PanCK (Pan-Cytokeratin) (purple) and HIF1A (brown; green arrowheads indicate double PanCK^+^-HIF1A^+^ tumor cells), PanCK (purple) and CD31 (brown), and CD34 (brown) (**E**), and quantification of EGFR and c-MET (tumor progression) (**F**, **G**), HIF1A (hypoxia and tumor invasion) (**H**), CD31 (undifferentiated blood vessels) (**I**) and CD34 (differentiated blood vessels) (**J**) positive areas in LLC-challenged *Tert*^*+/+*^ and G3 *Tert*^*−/−*^ mice. **K** Representative lung immunostainings for PanCK (purple) and γH2AX (brown; green arrowheads indicate double PanCK^+^-γH2AX^+^ tumor cells), p53 (brown, blue arrowheads indicate p53^+^ cells), PanCK (purple) and p21 (brown; red arrowheads indicate double PanCK^+^-p21^+^ tumor cells), PanCK (purple) and Cleaved Caspase-3 (C3, brown; black arrowheads indicate C3^+^ cells), and PanCK (purple) and Ki67 (brown; orange arrowheads indicate double PanCK^+^-Ki67^+^ tumor cells) in lung sections from LLC-challenged *Tert*^*+/+*^ and G3 *Tert*^*−/−*^ mice. Quantification of γH2AX and p53 (DNA damage response) (**L**–**M**), and p21 (cell cycle arrest) (**N**) positive areas, number of C3 (apoptosis) positive cells/mm^2^ (**O**) and Ki67 (proliferation) positive area (**P**) in LLC-challenged *Tert*^*+/+*^ and G3 *Tert*^*−/−*^ mice. Data are expressed as mean ± SEM (the number of mice is indicated in each case). *p < 0.05; **p < 0.01; ***p < 0.001 (Dunn–Sidak test for multiple comparisons and Mann–Whitney or unpaired *t* tests to compare 2 independent groups).

To determine the effect of TERT deficiency in the tumor microenvironment on lung tumor progression, invasion and vascularization, we assessed lung mRNA expression of *Mmp9*, *Hmox1* and *Egfr* (tumor progression) and *Hif1a* (hypoxia and tumor invasion), as well as performed immunostainings for EGFR and c-MET (tumor progression), and CD34 (differentiated blood vessels), and double immunostainings for the lung tumor cell marker PanCK (Pan-Cytokeratin) with HIF1A (hypoxia and tumor invasion) and CD31 (undifferentiated blood vessels) in lung sections of our mouse cohorts following induction of LLC (Fig. 2A–J). mRNA expression of *Mmp9, Hmox1*, *Egfr* and *Hif1a* showed a strong induction in LLC-challenged *Tert*^*+/+*^ mice, while this increment was attenuated in G3 *Tert*^*−/−*^ mice (Fig. 2A–D). In addition, G3 *Tert*^*−/−*^ mice showed decreased EGFR^+^, c-MET^+^, HIF1A^+^, CD31^+^ and CD34^+^ areas upon induction of LLC as compared to wild-type controls (Fig. 2E–J). Noteworthy, we observed a reduced presence of HIF1A^+^ tumor cells in G3 *Tert*^*−/−*^ mice compared to *Tert*^*+/+*^ mice (Fig. 2E).

To gain insight into how telomerase deficiency in the microenvironment protects from lung tumor implantation and progression, we next studied the impact of TERT deficiency on DNA damage response, cell cycle arrest, apoptosis and proliferation. To this end we performed double immunostainings for PanCK with γH2AX (DNA damage response), p21 (cell cycle arrest), Cleaved Capsase-3 (C3, apoptosis) and Ki67 (proliferation), as well as simple immunostainings for p53 (DNA damage response) in lung sections of our mouse cohorts upon induction of LLC (Fig. 2K–P). We found increased γH2AX^+^, p53^+^ and p21^+^ areas, as well as increased number of C3^+^ cells, and reduced Ki67^+^ area after induction of LLC in G3 *Tert*^*−/−*^ compared to *Tert*^*+/+*^ lungs (Fig. 2K–P). Of note, we observed reduced proliferation and increased numbers of lung tumor cells positive for DNA damage response and cell cycle arrest markers in *Tert*^*−/−*^ mice compared to wild-type controls (Fig. 2K).

### Telomerase deficiency in the tumor microenvironment decreases expression of lung inflammation and tumor immunosupression markers

In order to address the effect of TERT deficiency on peripheral blood cells following induction of LLC, we evaluated total and differential white blood cell counts, as well as circulating levels of the established clinical biomarkers TNF and IL6 [19] (Supplementary Fig. S3A–F). The proportion of circulating neutrophils and macrophages exhibited a marked increase in LLC-challenged *Tert*^*+/+*^ mice, which was not observed in G3 *Tert*^*−/−*^ mice (Supplementary Fig. S3B, D). Noteworthy, the lack of TERT expression did not change the proportion of circulating neutrophils and macrophages in non-LLC challenged G3 *Tert*^*−/−*^ mice compared to *Tert*^*+/+*^ mice (Supplementary Fig. S3B, D). In contrast, lymphocyte counts were found reduced only in *Tert*^*+/+*^ mice upon induction of LLC (Supplementary Fig. S3C). Moreover, IL6 and TNF levels in serum were increased in LLC-challenged *Tert*^*+/+*^ mice, while this increase was less pronounced in G3 *Tert*^*−/−*^ mice (Supplementary Fig. S3E, F). To further study the role of TERT deficiency in the lung tumor microenvironment, we quantified cellularity and total protein concentration in bronchoalveolar lavage fluid (BALF) (Supplementary Fig. S3G–L). Total and differential BALF cell counts for neutrophils, lymphocytes and macrophages were increased in *Tert*^*+/+*^ mice upon induction of LLC, while this increase was less pronounced in G3 *Tert*^*-/-*^ mice (Supplementary Fig. S3G–K). Total protein concentration in BALF, an indicator of vascular permeability [20–22], was significantly increased in LLC-challenged *Tert*^*+/+*^ mice, while these levels did not change in G3 *Tert*^*−/−*^ mice (Supplementary Fig. S3L).

**Fig. 3:**
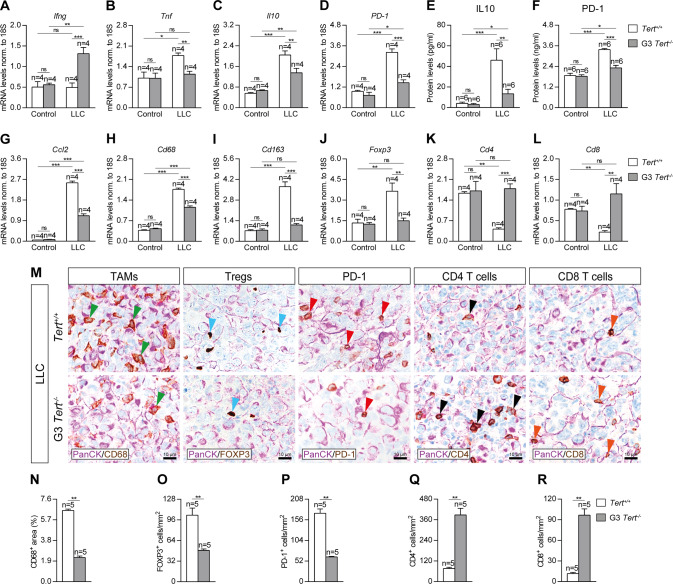
Telomerase deficiency in the tumor microenvironment decreases expression of lung inflammation and tumor immunosupression markers. Lung tissue mRNA expression levels of *Ifng* (anti-tumor immunity) (**A**), *Tnf* (Th1 inflammation) (**B**), and *Il10* and *PD-1* (tumor immunosupression) (**C**, **D**) normalized to 18S expression, and IL10 (**E**) and PD-1 (**F**) protein levels in lung homogenates from *Tert*^*+/+*^ and G3 *Tert*^*−/−*^ mice. Lung tissue mRNA expression levels of *Ccl2* (macrophage chemotaxis) (**G**), *Cd68* (tumor associated macrophages (TAMs)) (**H**), *Cd163* (M2 TAMs) (**I**), *Foxp3* (regulatory T cells (Tregs)) (**J**), *Cd4* (CD4^+^ helper T cells) (**K**) and *Cd8* (CD8^+^ cytotoxic T cells) (**L**) normalized to 18S expression in *Tert*^*+/+*^ and G3 *Tert*^*−/−*^ mice. Representative lung immunostainings for PanCK (purple) with CD68 (brown; green arrowheads indicate CD68^+^ cells), FOXP3 (brown; blue arrowheads indicate FOXP3^+^ cells), PD-1 (brown; red arrowheads indicate PD-1^+^ cells), CD4 (brown; black arrowheads indicate CD4^+^ cells), and CD8 (brown; orange arrowheads indicate CD8^+^ cells) (**M**), and quantification of CD68 positive area (**N**), and number of FOXP3 (**O**), PD-1 (**P**), CD4 (**Q**) and CD8 (**R**) positive cells/mm^2^ in LLC-challenged *Tert*^*+/+*^ and G3 *Tert*^*−/−*^ mice. Data are expressed as mean ± SEM (the number of mice is indicated in each case). *p < 0.05; **p < 0.01; ***p < 0.001 (Dunn–Sidak test for multiple comparisons and Mann–Whitney or unpaired t tests to compare 2 independent groups).

To evaluate the implication of TERT deficiency on lung tissue inflammation and immunosupression, we assessed lung mRNA expression and protein levels of inflammation and immunosuppression markers on lung homogenates of our mouse cohorts by qPCR and ELISA (Fig. 3A–L). First, mRNA expression of *Ifng* (anti-tumor immunity) was significantly increased only in G3 *Tert*^*−/−*^ mice after LLC challenge (Fig. 3A). *Tnf* (Th1 inflammation) mRNA levels were found significantly increased in *Tert*^*+/+*^ mice upon induction of LLC, but did not change in LLC-challenged G3 *Tert*^*−/−*^ mice (Fig. 3B). Concerning immunosupression markers, *Il10* and *PD-1* mRNA levels were augmented in *Tert*^*+/+*^ mice after LLC challenge, whilst this increase was lower in G3 *Tert*^*−/−*^ mice (Fig. 3C, D). Accordingly, IL10 and PD-1 protein levels assessed by ELISA were also higher in LCC challenged wild-type than in G3 *Tert*^*−/−*^ mice (Fig. 3E, F). Regarding inflammation, mRNA expression of *Ccl2* (macrophage chemotaxis), *Cd68* (tumor associated macrophages, TAMS), *Cd163* (M2 TAMs) and *Foxp3* (regulatory T cells, Tregs) markers was greatly induced upon LLC challenge in *Tert*^*+/+*^ mice, while this increment was lower and specifically unaltered in the case of *Cd163* and *Foxp3*, in G3 *Tert*^*−/−*^ mice (Fig. 3G–J). Interestingly, mRNA expression of *Cd4* (CD4^+^ T cells) and *Cd8* (CD8^+^ T cells) markers showed a sharp drop in LLC-challenged *Tert*^*+/+*^ mice that was not detected in G3 *Tert*^*−/−*^ mice (Fig. 3K, L). On the other hand, to complement this data we performed double immunostainings for PanCK with CD68, FOXP3, PD-1, CD4 and CD8 in LLC-challenged lungs (Fig. 3M–R). We found a reduced presence of CD68^+^, FOXP3^+^, PD-1^+^ cells, along with an increased number of CD4^+^ and CD8^+^ cells in the lungs of LLC-challenged G3 *Tert*^*−/−*^ with respect to *Tert*^*+/+*^ mice (Fig. 3M–R). These findings indicate that TERT may promote lung inflammation and immunosuppression.

### Telomerase deficiency attenuates lung tumor invasion by reducing epithelial to mesenchymal transition and fibrosis

**Fig. 4:**
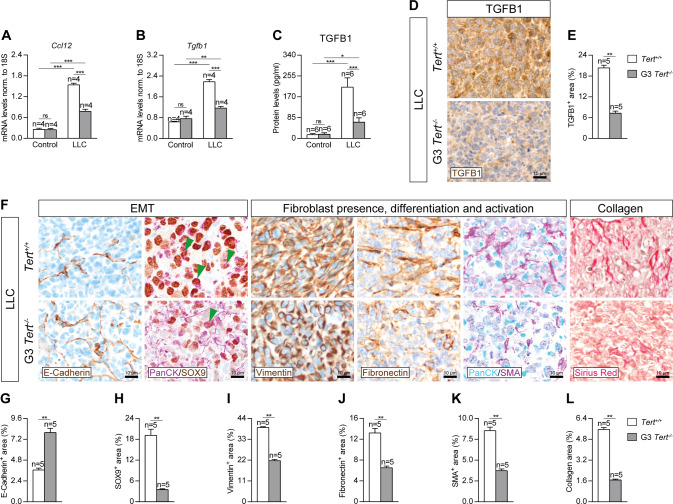
Telomerase deficiency attenuates lung tumor invasion by reducing epithelial to mesenchymal transition and fibrosis. Lung tissue mRNA expression levels of *Ccl12* (recruitment of fibrocytes) (**A**), *Tgfb1* (EMT and tumor invasion) (**B**) normalized to 18S expression, and TGFB1 (**C**) protein levels in lung homogenates from *Tert*^*+/+*^ and G3 *Tert*^*−/−*^ mice. Representative lung immunostainings for TGFB1 (brown) (**D**) and quantification of TGFB1^+^ areas in LLC-challenged *Tert*^*+/+*^ and G3 *Tert*^*−/−*^ mice (**E**). Representative lung immunostainings for E-Cadherin (brown), PanCK (purple) and SOX9 (brown; green arrowheads indicate double PanCK^+^-SOX9^+^ tumor cells), Vimentin (brown), Fibronectin (brown), PanCK (blue) and SMA (purple), and Sirius Red staining (**F**), and quantification of E-Cadherin (**G**), SOX9 (**H**), Vimentin (**I**), Fibronectin (**J**), SMA (**K**) and Collagen (Sirius Red) (**L**) positive areas in LLC-challenged *Tert*^*+/+*^ and G3 *Tert*^*−/−*^ mice. Data are expressed as mean ± SEM (the number of mice is indicated in each case). *p < 0.05; **p < 0.01; ***p < 0.001 (Dunn–Sidak test for multiple comparisons and Mann–Whitney or unpaired t tests to compare 2 independent groups).

To study how TERT deficiency protects from lung tumor invasion, we performed a qPCR to assess mRNA expression of *Ccl12* (recruitment of fibrocytes) and *Tgfb1* (epithelial-mesenchymal transition (EMT) and tumor invasion) markers, an ELISA to quantify TGFB1 protein levels on lung homogenates of our mouse cohorts, as well as immunostainings for TGFB1 in lung sections of LLC-challenged mice (Fig. 4A–E). *Ccl12* and *Tgfb1* mRNA expression was greatly increased in *Tert*^*+/+*^ mice after LLC challenge, while this increase was milder in G3 *Tert*^*−/−*^ mice (Fig. 4A, B). In accordance, *Tert*^*−/−*^ mice exhibited reduced TGFB1 protein levels, and expression (TGFB1^+^ area) (Fig. 4C–E).

Next, to further confirm that TERT deficiency confers resistance to lung tumor invasion, we performed immunostainings for the epithelial to mesenchymal transition (EMT) marker E-Cadherin, and for the fibroblast presence and differentiation markers Vimentin and Fibronectin, double immunostainings for PanCK with the EMT and fibroblast activation markers SOX9 and SMA (Smooth Muscle Actin), as well as a Sirius Red staining to assess collagen deposition in lung sections of LLC-challenged mice (Fig. 4F–L). E-Cadherin exhibited an increased stained area in *Tert*^*−/−*^ lungs compared to *Tert*^*+/+*^ mice. Conversely, SOX9, Vimentin, Fibronectin, SMA and Sirius Red showed reduced stained areas in G3 *Tert*^*−/−*^ compared to *Tert*^*+/+*^ lungs (Fig. 4F–L). Notably, we observed a reduced presence of double PanCK^+^-SOX9^+^ tumor cells in G3 *Tert*^*−/−*^ mice with respect to *Tert*^*+/+*^ mice (Fig. 4F).

### Telomere dysfunction induced by 6-thio-dG treatment decreases lung tumor implantation

**Fig. 5:**
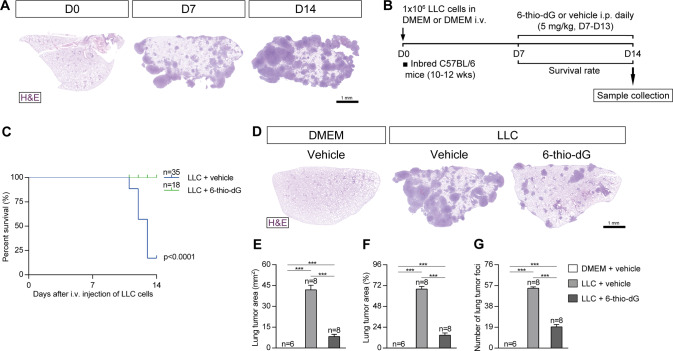
Telomere dysfunction induced by 6-thio-dG treatment decreases lung tumor implantation. **A** Representative images (H&E) of lungs showing LLC implantation at different time points in inbred C57BL/6 mice. **B** Protocol for the induction of Lewis Lung Carcinoma (LLC) in inbred C57BL/6 mice. 1 × 10^6^ Lewis cells suspended in 100 µl of DMEM or equal volume of DMEM (controls) were injected via the tail vein of 10–12 weeks old inbred C57BL/6 male mice on day 0 (D0) and daily intraperitoneal (i.p.) injections of 6-thio-dG (5 mg/kg) or vehicle were administered once the LLC was established (D7-D14), as well as a follow up of survival until sample collection on D14. Kaplan–Meier survival curves (**C**), representative images of LLC-challenged mice treated with 6-thio-dG vs. control lungs (H&E) (**D**), and quantification of lung tumor area (**E**, **F**) and foci (**G**). Data are expressed as mean ± SEM (the number of mice is indicated in each case). ***p < 0.001 (Dunn–Sidak test for multiple comparisons).

Since TERT deficiency impairs lung tumor implantation, invasion and progression, we next set to investigate this phenomena following induction of dysfunctional telomeres in a NSCLC mouse model. To this end, we induced a telomere dysfunction in inbred C57BL/6 mice by administration of the telomerase substrate precursor 6-thio-dG, known to induce telomere dysfunction [16, 21] at the time the LLC was established at day (D) 7 (Fig. 5A, B). We generated the following experimental groups: DMEM + vehicle (DMEM i.v. + 5% DMSO i.p. daily between D7 and D14); LLC + vehicle (LLC i.v + 5% DMSO i.p. daily between D7 and D14); and LLC + 6-thio-dG (LLC i.v. + 5 mg/kg of 6 thio-dG in 5% DMSO i.p. daily between D7 and D14).

First, we determined the effect of telomere dysfunction on survival and lung tumor implantation. For this purpose we assessed survival, lung tumor area and number of tumor foci (Fig. 5C–G). Remarkably, 6-thio-dG-treated mice showed a survival of 100% upon induction of LLC with respect to LLC control mice, which did not survive the LLC challenge (Fig. 5C). 6-thio-dG-treated mice also exhibited reduced tumor implantation with respect to LLC control mice as indicated by decreased number of lung tumor area and foci (Fig. 5D–G).

### Telomere dysfunction mediated by 6-thio-dG reduces lung tumor progression, invasion and vascularization, increases telomeric damage, cell cycle arrest and apoptosis, and reduces proliferation

**Fig. 6:**
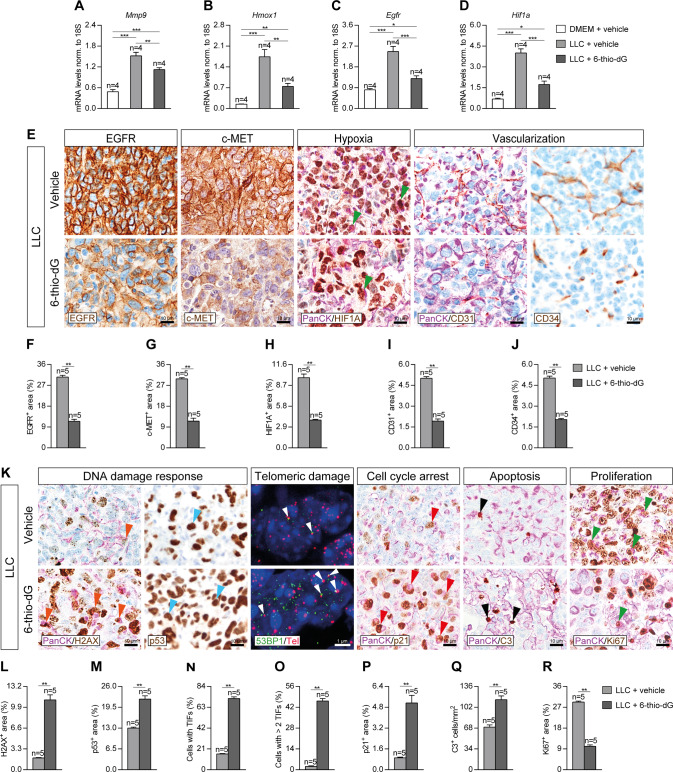
Telomere dysfunction mediated by 6-thio-dG reduces lung tumor progression, invasion and vascularization, increases telomeric damage, cell cycle arrest and apoptosis, and reduces proliferation. Lung tissue mRNA expression levels of *Mmp9* (**A**), *Hmox1* (**B**), *Egfr* (**C**) (tumor progression markers) and *Hif1a* (hypoxia and tumor invasion) (**D**) normalized to 18S expression in LLC-challenged mice treated with 6-thio-dG vs. controls. Representative lung immunostainings for EGFR (brown), c-MET (brown), PanCK (Pan-Cytokeratin) (purple) and HIF1A (brown; green arrowheads indicate double PanCK^+^-HIF1A^+^ tumor cells), PanCK (purple) and CD31 (brown), and CD34 (brown) (**E**), and quantification of EGFR and c-MET (tumor progression) (**F**, **G**), HIF1A (hypoxia and tumor invasion) (**H**), CD31 (undifferentiated blood vessels) (**I**) and CD34 (differentiated blood vessels) (**J**) positive areas in mice treated with 6-thio-dG or vehicle upon LLC challenge. **K** Representative lung immunostainings for PanCK (purple) and γH2AX (brown; orange arrowheads indicate double PanCK^+^-γH2AX^+^ tumor cells), p53 (brown, blue arrowheads indicate p53^+^ cells), PanCK (purple) and p21 (brown; red arrowheads indicate double PanCK^+^-p21^+^ tumor cells), PanCK (purple) and Cleaved Caspase-3 (C3, brown; black arrowheads indicate C3^+^ cells), and PanCK (purple) and Ki67 (brown; red arrowheads indicate double PanCK^+^-Ki67^+^ tumor cells), and immuno-telomere-Q-FISH with the DNA damage response marker 53BP1 to assess telomeric induced foci (TIFs) (white arrowheads) in lung sections from mice treated with 6-thio-dG or vehicle upon LLC challenge. Quantification of γH2AX and p53 (DNA damage response) positive areas (**L**, **M**), percentage of cells with TIFs or more than 2 TIFs (**N**, **O**), and p21 (cell cycle arrest) (**P**) positive areas, number of C3 (apoptosis) positive cells/mm^2^ (**Q**) and Ki67 (proliferation) positive area (**R**) in mice treated with 6-thio-dG or vehicle upon LLC challenge. Data are expressed as mean ± SEM (the number of mice is indicated in each case). *p < 0.05; **p < 0.01; ***p < 0.001 (Dunn–Sidak test for multiple comparisons and Mann–Whitney or unpaired *t* tests to compare 2 independent groups).

Next, to determine the implication of dysfunctional telomeres on lung tumor progression, invasion and vascularization, we assessed lung mRNA expression of *Mmp9*, *Hmox1* and *Egfr* (tumor progression markers) and *Hif1a* (hypoxia and tumor invasion marker), as well as performed immunostainings for EGFR and c-MET (tumor progression markers), and CD34 (marker of differentiated blood vessels), and double immunostainings for the lung tumor cell marker PanCK with HIF1A (hypoxia and tumor invasion markers) and CD31 (undifferentiated blood vessels marker) in lung sections of our mouse cohorts after induction of LLC (Fig. 6A–J). Interestingly, mRNA expression of *Mmp9*, *Hmox1*, *Egfr*, and *Hif1a* was significantly decreased in 6-thio-dG-treated as compared to control mice (Fig. 6A–D). Furthermore, 6-thio-dG-treated mice exhibited diminished EGFR^+^, c-MET^+^, HIF1A^+^, CD31^+^ and CD34^+^ areas with respect to LLC control mice (Fig. 6E–J). Of note, we observed a decreased presence of HIF1A^+^ tumor cells in 6-thio-dG-treated mice with respect to LLC control mice (Fig. 6E).

To further investigate how dysfunctional telomeres protect from lung tumor implantation and progression, we performed double immunostainings for PanCK with γH2AX (DNA damage response marker), p21 (cell cycle arrest marker), Cleaved Caspase 3 (C3, apoptosis marker) and Ki67 (proliferation marker), simple immunostainings for p53 (DNA damage response marker), as well as an immuno-telomere-Q-FISH with the DNA damage response marker 53BP1 to evaluate telomeric induced foci (TIFs) in lung sections of our mouse cohorts upon induction of LLC (Fig. 6K–R). Interestingly, 6-thio-dG-treated mice showed increased γH2AX^+^, p53^+^ and p21^+^ areas, increased number of C3^+^ cells, as well as a higher proportion of cells with TIFs compared to LLC control mice (Fig. 6K–Q). Conversely, 6-thio-dG-treated mice also showed a reduced Ki67^+^ area (Fig. 6K, R). Specifically, we observed an increased presence of lung tumor cells positive for DNA damage response and cell cycle arrest markers, as well as reduced presence of proliferating tumor cells in 6-thio-dG-treated mice compared to LLC controls (Fig. 6K). Of note, normal lung tissues in non-LLC challenged mice (DMEM + vehicle) do not show induction of DNA damage response, cell cycle arrest, apoptosis and proliferation since very few if any positive cells for H2AX, p21, p53, C3 and Ki67 were detected (Supplementary Fig. S4).

### Telomere dysfunction induced by 6-thio-dG treatment reduces expression of lung inflammation and tumor immunosupression markers

In order to evaluate the effect of telomere dysfunction induced by 6-thio-dG on peripheral blood following induction of LLC, we evaluated total and differential white blood cell counts, as well as circulating levels of TNF and IL6 (Supplementary Fig. S5A–F). We found increased total and differential blood cell counts for neutrophils and macrophages in LLC control mice, while this increase was not observed in 6-thio-dG-treated mice (Supplementary Fig. S5A, B, D). Notably, total and neutrophil counts were even lower in 6-thio-dG-treated than in DMEM control mice (Supplementary Fig. S5A, B). There were no significant changes in lymphocyte counts among the different mouse cohorts (Supplementary Fig. S5C). In addition, IL6 and TNF levels in serum were strongly induced in LLC control mice, a phenomenon not observed in 6-thio-dG-treated mice (Supplementary Fig. S5E, F). Moreover, to further study the implication of dysfunctional telomeres in the lung tumor microenvironment, we quantified cellularity and total protein concentration in BALF (Supplementary Fig. S5G–L). Total and differential cell counts, as well as total protein concentration in BALF were found significantly decreased in 6-thio-dG-treated mice compared to control mice (Supplementary Fig. S5G–L).

**Fig. 7:**
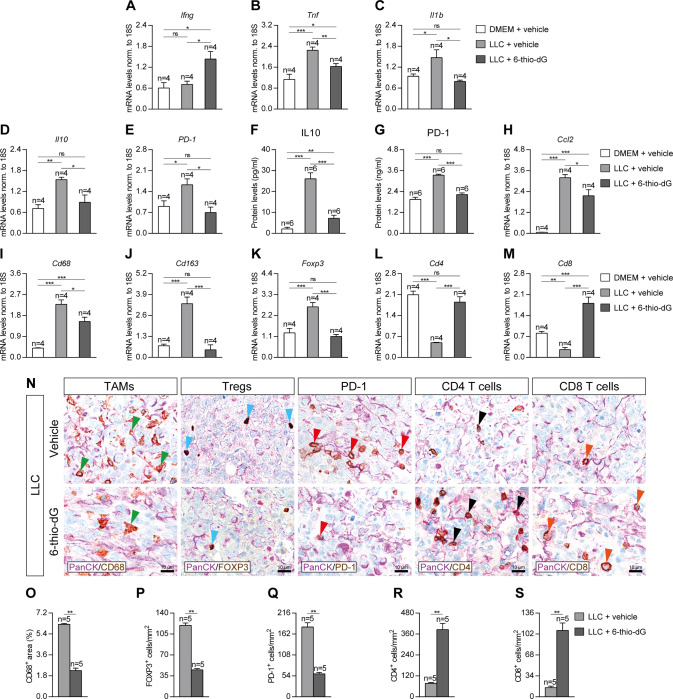
Telomere dysfunction induced by 6-thio-dG treatment reduces expression of lung inflammation and tumor immunosupression markers. Lung tissue mRNA expression levels of *Ifng* (anti-tumor immunity) (**A**), *Tnf* and *Il1b* (Th1 inflammation) (**B**, **C**) and *Il10* and *PD-1* (tumor immunosupression) (**D**, **E**) normalized to 18S expression, and IL10 (**F**) and PD-1 (**G**) protein levels in lung homogenates from LLC-challenged mice treated with 6-thio-dG vs. controls. Lung tissue mRNA expression levels of *Ccl2* (macrophage chemotaxis) (**H**), *Cd68* (tumor associated macrophages (TAMs)) (**I**), *Cd163* (M2 TAMs) (**J**), *Foxp3* (regulatory T cells (Tregs)) (**K**), *Cd4* (CD4^+^ helper T cells) (**L**) and *Cd8* (CD8^+^ cytotoxic T cells) (**M**) normalized to 18S expression in LLC-challenged mice treated with 6-thio-dG vs. controls. Representative lung immunostainings for PanCK (purple) with CD68 (brown; green arrowheads indicate CD68^+^ cells), FOXP3 (brown; blue arrowheads indicate FOXP3^+^ cells), PD-1 (brown; red arrowheads indicate PD-1^+^ cells), CD4 (brown; black arrowheads indicate CD4^+^ cells), and CD8 (brown; orange arrowheads indicate CD8^+^ cells) (**N**), and quantification of CD68 positive area (**O**), and number of FOXP3 (**P**), PD-1 (**Q**), CD4 (**R**) and CD8 (**S**) positive cells/mm^2^ in LLC-challenged mice treated with 6-thio-dG vs. controls. Data are expressed as mean ± SEM (the number of mice is indicated in each case). *p < 0.05; **p < 0.01; ***p < 0.001 (Dunn–Sidak test for multiple comparisons and Mann–Whitney or unpaired *t* tests to compare 2 independent groups).

To assess the implication of dysfunctional telomeres on lung tissue inflammation and immunosupression, we assessed lung mRNA expression and protein levels of inflammation and immunosuppression markers on lung homogenates of our mouse cohorts by qPCR and ELISA (Fig. 7A–M). Interestingly, mRNA expression of *Ifng* (anti-tumor immunity marker) was only found significantly increased in 6-thio-dG-treated mice (Fig. 7A). In addition, mRNA levels of *Tnf* and *Il1b* (Th1 inflammation marker) were significantly increased in LLC control mice, while this increment was milder for *Tnf* and not observed in the case of *Il1b* in 6-thio-dG-treated mice (Fig. 7B, C). Regarding mRNA expression of immunosupression markers, the strong induction of *Il10* and *PD-1* observed in LLC control mice, was not shown by 6-thio-dG-treated mice (Fig. 7D, E). Accordingly, IL10 and PD-1 protein levels assessed by ELISA supported mRNA expression profile (Fig. 7F, G). On the other hand, mRNA expression of *Ccl2* (macrophage chemotaxis marker), *Cd68* (tumor associated macrophages, TAMs), *Cd163* (M2 TAMs) and *Foxp3* (regulatory T cells, Tregs) markers was greatly increased in LLC control mice, while this increment was less pronounced in 6-thio-dG-treated mice (Fig. 7H–K). Remarkably, mRNA expression of *Cd4* (CD4^+^ T cells) and *Cd8* (CD8^+^ T cells) markers was strongly reduced in LLC control mice, but not in 6-thio-dG-treated mice, which even showed increased expression of *Cd8* levels compared to DMEM and LLC control mice (Fig. 7L, M). Furthermore, we performed double immunostainings for PanCK with CD68, FOXP3, PD-1, CD4 and CD8 in LLC-challenged lungs (Fig. 7N–S). We found a reduced presence of CD68^+^, FOXP3^+^ and PD-1^+^ cells, along with an increased number of CD4^+^ and CD8^+^ cells in 6-thio-dG-treated mice with respect to LLC control mice (Fig. 7N–S). These results indicate that 6-thio-dG treatment reduces lung inflammation and immunosuppression.

### Telomere dysfunction mediated by 6-thio-dG attenuates lung tumor invasion by reducing epithelial to mesenchymal transition and fibrosis, and reduces tumor growth in NSCLC patient-derived xenografts

**Fig. 8:**
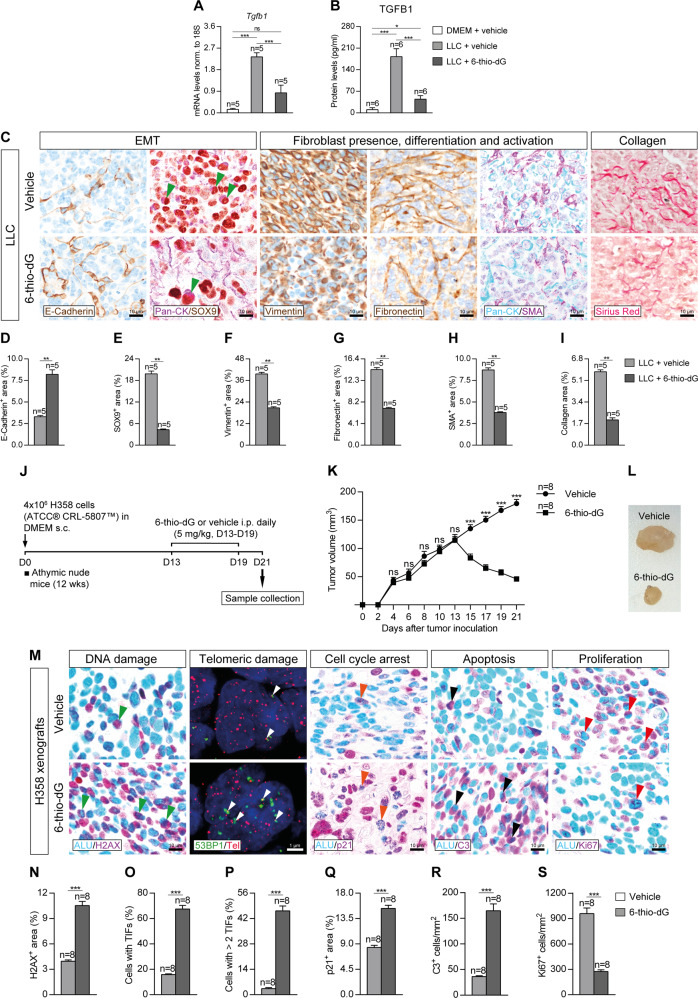
Telomere dysfunction mediated by 6-thio-dG attenuates lung tumor invasion by reducing epithelial to mesenchymal transition and fibrosis, and reduces tumor growth in NSCLC patient-derived xenografts. Lung tissue mRNA expression levels of *Tgfb1* (EMT and tumor invasion) (**A**) normalized to 18 S expression, and TGFB1 (**B**) protein levels in lung homogenates from mice treated with 6-thio-dG or vehicle upon LLC challenge. Representative lung immunostainings for E-Cadherin (brown), PanCK (purple) and SOX9 (brown; green arrowheads indicate double PanCK^+^-SOX9^+^ tumor cells), Vimentin (brown), Fibronectin (brown), PanCK (blue) and SMA (purple), and Sirius Red staining (**C**), and quantification of E-Cadherin (**D**), SOX9 (**E**), Vimentin (**F**), Fibronectin (**G**), SMA (**H**) and Collagen (Sirius Red) (**I**) positive areas in mice treated with 6-thio-dG or vehicle upon LLC challenge. **J** Protocol for the generation of the human H358 NSCLC Xenofraft model. 4 × 10^6^ H358 cells suspended in 100 µl of RPMI were subcutaneously injected in the right flank of 10–12 weeks old athymic nude male on day 0 (D0), and daily intraperitoneal injections of 6-thio-dG (5 mg/kg) or vehicle (5% DMSO) were administered once the tumor reached 100 mm^3^ (D13-D19). **K** A follow-up of the tumor growth was performed by measuring the tumor volume and H358 xenografts were resected on D21. **L** Macroscopic image of representative H358 xenograts from animals treated with 6-thio-dG or vehicle. **M** Representative lung immunostaining for ALU (blue) and γH2AX (purple; green arrowheads indicate double ALU^+^-γH2AX^+^ tumor cells), immuno-telomere-Q-FISH with the DNA damage response marker 53BP1 to assess telomeric induced foci (TIFs) (white arrowheads), and lung immunostainings for ALU (blue) and p21 (purple; orange arrowheads indicate double ALU^+^-p21^+^ tumor cells), ALU (blue) and C3 (purple; black arrowheads indicate double ALU-C3^+^ cells), and ALU (blue) and Ki67 (purple; red arrowheads indicate double ALU^+^-Ki67^+^ tumor cells) in H358 Xenografts from animals treated with 6-thio-dG or vehicle. Quantification of γH2AX (DNA damage response) positive area (**N**) percentage of cells with TIFs or more than 2 TIFs (**O**, **P**), p21 (cell cycle arrest) positive area (**Q**), and number of C3 (apoptosis) (**R**) and Ki67 (proliferation) (**S**) cells/mm^2^ in H358 Xenograts from animals treated with 6-thio-dG or vehicle. Data are expressed as mean ± SEM (the number of mice is indicated in each case). *p < 0.05; **p < 0.01; ***p < 0.001 (Dunn–Sidak test for multiple comparisons and Mann–Whitney or unpaired *t* tests to compare 2 independent groups).

To further study how telomere dysfunction induced by 6-thio-dG protects from lung tumor invasion, we performed both qPCR and ELISA to assess mRNA and protein levels of TGFB1 in lung homogenates, and immunostainings for E-Cadherin (EMT marker), Vimentin and Fibronectin (markers of fibroblast presence and differentiation), and double immunostainings for PanCK with SOX9 (EMT marker) and SMA (Smooth Muscle Actin) (fibroblast activation marker), as well as a Sirius Red staining to assess collagen deposition in lung sections of LLC-challenged mice (Fig. 8A–I). In particular, *Tgfb1* mRNA expression and TGFB1 protein levels were found greatly increased in LLC control mice, while this increase was no observed in 6-thio-dG-treated mice (Fig. 8A, B). Accordingly, 6-thio-dG-treated mice exhibited increased expression of E-Cadherin as compared to LLC control mice. In contrast, SOX9, Vimentin, Fibronectin, SMA and Sirius Red showed reduced stained areas in 6-thio-dG-treated mice compared to LLC control mice (Fig. 8C–I). Of note, we observed a reduced presence of double PanCK^+^-SOX9^+^ tumor cells in 6-thio-dG-treated mice compared to LLC control mice (Fig. 8C).

In order to test the anti-tumor activity of 6-thio-dG, we performed a human NSCLC xenograft model. For that purpose, H358 cells isolated from a human bronchioalveolar carcinoma (NCI-H358 [H-358, H358] (ATCC® CRL-5807™)) were subcutaneously injected in athymic nude mice. When the tumor volume reached 100 mm^3^, 6-thio-dG was intraperitoneally administered (Fig. 8J). Tumor volume was longitudinally measured for 21 days (Fig. 8K). In this respect, H358 xenografts from 6-thio-dG-treated mice showed reduced tumor growth compared with vehicle-treated animals (Fig. 8K, L). We also performed double immunostainings for the human ALU marker with γH2AX (DNA damage response marker), p21 (cell cycle arrest marker), C3 (apoptosis marker) and Ki67 (proliferation marker), as well as an immuno-telomere-Q-FISH with the DNA damage response marker 53BP1 to evaluate TIFs on tissue sections of H358 Xenografts (Fig. 8M–S). H358 xenografts from 6-thio-dG-treated mice demonstrated increased presence of H2AX, p21 and C3 positive cells, along with a higher proportion of cells with TIFs. By contrast, 6-thio-dG-treated mice also exhibited a reduced number of Ki67 positive cells (Fig. 8M, S). Of note, we observed an increased presence of H358 tumor cells with DNA damage, cell cycle arrest and apoptosis, as well as reduced presence of proliferating H358 tumor cells in 6-thio-dG-treated mice compared to wild-type controls (Fig. 8M).

## Discussion

Tumor progression depends on interactions between cancer cells and the tumor microenvironment. In the past, it has been suggested that TERT could be modulating the tumor microenvironment [3, 12]. To study the potential role of TERT in the lung tumor microenvironment, we generated NSCLC models by inducing a Lewis Lung Carcinoma (LLC) in wild-type and TERT-deficient mice. In both murine and human xenograph models, we also tested the anti-tumor activity of the nucleoside analog 6-thio-dG, which is incorporated into telomeric DNA, leading to telomere dysfunction [16]. We first explored genomic alterations in the *TERT* locus and their correlation with survival in NSCLC patients, as well as the correlation of *TERT* with immune infiltrates.

We report increased copy number and mRNA expression of TERT in NSCLC patients, which could be contributing to a lower survival. Accordingly, TERT activity has been reported in tumoral lung tissues from NSCLC patients and was therefore proposed as a bad prognostic factor in NSCLC [23–25]. Also in line with this, TERT has been found overexpressed and mutated in multiple human cancers, as well as in mouse tumors [12, 13, 17]. Increased TERT expression in NSCLC was found specifically associated to a reduced infiltration of CD8^+^ T lymphocytes, as well as to an increased infiltration of MDSCs. Noteworthy, activation of CD8^+^ T cells in NSCLC correlates with a stronger antitumor immunity [26], while infiltration of MDSCs was found associated to a poor prognosis in NSCLC patients [27].

Interestingly, we found that TERT deficiency in the tumor microenvironment decreased TERT activity and expression in LLC tumor samples as compared with telomerase proficient microenvironment. Moreover, both TERT deficiency and dysfunctional telomeres decreased lung tumor implantation and expression of the tumor progression markers *Mmp9*, *Egfr*, *Hmox1* and c-MET. In this regard, TERT inhibition and dysfunctional telomeres were shown to reduce K-Ras-induced lung tumor growth in mice [28]. Of note, MMP9 was reported to promote LLC cell invasiveness in mice [29]. MMP9, EGFR and c-MET, were found overexpressed in NSCLC patients and associated with a poor prognosis [30–33]. In addition, HMOX1 was reported to promote lung tumorigenesis in mice, and its high expression was correlated with tumor invasiveness in NSCLC [34, 35]. On the other hand, TERT deficiency and dysfunctional telomeres, resulted in reduced hypoxia, vascularization and vascular permeability by decreased expression of HIF1A, CD31 and CD34, as well as by reduced total protein concentration in BALF, a well-established indicator of vascular permeability [20–22]. In accordance, TERT deficiency was reported to inhibit vascular development in Lewis Lung Carcinoma xenografts [36]. Specifically, in response to hypoxia, HIF-1α is overexpressed and/or activated and targets those genes which are required for angiogenesis, metabolic adaptation to low oxygen and promotes tumor survival [37–39]. Noteworthy, assessment of undifferentiated (CD31^+^) and differentiated (CD34^+^) blood vessels is an important prognostic factor in advanced NSCLC [40, 41]. It should be noted that reduced vascularization observed upon TERT deficiency and dysfunctional telomeres indicates that the ability of tumor cells to extravasate could be reduced in G3 *Tert*^*-/-*^ and 6-thio-dG treated mice. Our results indicate that TERT has a role in promoting lung tumor implantation and progression. Our findings also indicate that dysfunctional telomeres induced by 6-thio-dG treatment are also key to counteract tumor growth.

Notably, TERT deficiency and dysfunctional telomeres reduced peripheral IL6 and TNF levels, as well as expression of inflammation and tumor immunosuppression markers *Tnf*, *Ifng*, IL10 and PD-1 upon LLC challenge. Interestingly, elevated serum IL6 and TNF levels were found associated with tumor recurrence in NSCLC patients [19]. Noteworthy, TERT deficiency and dysfunctional telomeres in mice were shown to confer resistance to initiation of the inflammatory response [21, 42]. In particular, 6-thio-dG was recently reported to trigger anti-tumor immunity via interferon signaling, activating CD8^+^ T cells [43]. TNF was found elevated in the tumor microenvironment of NSCLC patients and was suggested to promote EMT, invasion and metastasis in NSCLC [19, 44]. IL10 was shown to contribute to tumor aggressiveness and poor survival in NSCLC, and PD-1 was demonstrated to be a key element in tumor-immune resistance [45–48]. We found that TERT deficiency and dysfunctional telomeres decreased presence of CD68^+^ TAMs and FOXP3^+^ Tregs, and increased CD4^+^ and CD8^+^ numbers upon induction of LLC. Of note, many cells modulate the antitumor response including TAMs, FOXP3^+^ Tregs, CD4^+^ helper T cells and CD8^+^ cytotoxic T cells [47]. Specifically, the tumor microenvironment of NSCLC contains a large amount of TAMs, which influence tumor progression. In this regard, accumulation of M2 TAMs was found associated to hypoxia, as well as with the production of IL-10 and TGFβ [49, 50]. Moreover, FOXP3 was reported to promote tumor growth by inducing EMT in NSCLC [51]. By contrast, activation of CD8^+^ and CD4^+^ T cells in NSCLC correlates with a stronger antitumor immunity [26, 52–54]. It should be noted that assessment of activated vs. exhausted T cells could help to confirm anti-tumor immunity in both TERT-deficient and 6-thio-dG treated mice. Our results indicate that TERT deficiency and dysfunctional telomeres confer resistance to initiation of lung tumor-associated inflammation and immunosupression.

TERT deficiency and dysfunctional telomeres also decreased TGFB expression, EMT and fibrosis upon LLC challenge. Particularly, exoxomes isolated from serum of lung cancer patients were reported to express TERT and transform nonmalignant fibroblasts into TERT positive cells [55]. Of note, TGFβ signaling was shown to promote EMT, which is manifested by the loss of E-Cadherin and increased expression of SOX9 and Vimentin, thus promoting tumor invasion in NSCLC [56–60]. Furthermore, smooth muscle actin (SMA), a clasical marker of cancer-associated fibroblasts, and fibronectin were shown to stimulate NSCLC growth [61, 62]. On the other hand, TERT deficiency and dysfunctional telomeres increased DNA and telomeric damage, cell cycle arrest and apoptosis, and reduced proliferation upon LLC and H358 challenges, contributing to a reduced tumor growth. Accordingly, TERT inhibition has shown to reduce K-Ras-induced lung tumor growth in mice by increased DNA damage and apoptosis [28]. This phenomena were previously reported in tumor cells upon treatment with 6-thio-dG [63–65].

In vivo toxicity testing of 6-thio-dG at effective doses did not reveal any significant hematologic, renal, or gastrointestinal system rate-limiting side effects [64]. One limitation of our study is that we had a short window of time to perform 6-thio-dG treatments in mice, which is in accordance with a prior study in which we observed that mice experienced an overdose if the treatment was extended for more than 10 days using the same dose (5 mg/kg) and administration protocol [21]. In addition, LLC tumors are very aggressive and animals do not live longer than 14 days. In agreement with previous works, our results support that 6-thio-dG could constitute a potential therapeutic approach in NSCLC [63, 64]. In accordance, 6-thio-dG was recently shown to enhance radiosensitivity in NSCLC [66].

In summary, we address by the first time the implication of TERT and dysfunctional telomeres in the lung tumor microenvironment. Our results demonstrate that targeting telomeres might be an effective therapeutic strategy in NSCLC.

## Materials and methods

### Data from NSCLC patients

Genomic data on amplification frequency, mRNA expression and copy number values of *TERT* in tissue samples from NSCLC patients, were obtained from the cBio Cancer Genomics Portal (cBioPortal) [67]. Survival curves of NSCLC patients and *TERT* mRNA expression in different types of cancers were obtained from the Kaplan–Meier Plotter [68] and TIMER 2.0 databases [69], which allow access to gene expression profiles and association with survival from The Cancer Genome Atlas (TCGA), Gene Expression Omnibus (GEO) and European Genome-Phenome Archive (EGA) databases. Association between immune infiltrates and expression of *TERT* was obtained from the TIMER 2.0 database. For additional details see online supplementary methods.

### Lewis lung carcinoma (LLC) models

*Tert* heterozygous mice were generated as previously described [70] and backcrossed to >98% C57BL/6 background. *Tert*^*+/+*^ and third (G3) generation *Tert*^*−/−*^ male mice were generated as illustrated in Fig. 1G. On day (D0), 1 × 10^6^ Lewis cells (LL/2 (LLC1) (ATCC® CRL-1642™)) suspended in 100 µl of DMEM or equal volume of DMEM (controls) were injected via the tail vein [71] of 10–12 weeks old *Tert*^*+/+*^ and G3 *Tert*^*−/−*^ male mice (Fig. 1H). A second LLC model was generated using 10–12 weeks old inbred C57BL/6 male mice (Charles River Laboratories, Wilmington, MA) injected via the tail vein with 1 × 10^6^ Lewis cells suspended in 100 µl of DMEM or equal volume of DMEM, along with daily intraperitoneal injections of 6-thio-dG (5 mg/kg) [21] or vehicle, once the LLC was established at D7 (D7-D14) (Fig. 5A, B). The following experimental groups were generated: DMEM + vehicle (DMEM i.v. +5% DMSO i.p. daily between D7 and D14); LLC + vehicle (LLC i.v + 5% DMSO i.p. daily between D7 and D14) and LLC + 6-thio-dG (LLC i.v. + 5 mg/kg of 6 thio-dG in 5% DMSO i.p. daily between D7 and D14). In parallel, an in vivo follow-up of survival was performed in both models. For additional details see online supplementary methods.

### Human NSCLC xenograft model

On day (D0), 4 × 10^6^ H358 cells (human bronchoalveolar carnicoma; NCI-H358 [H-358, H358] (ATCC® CRL-5807™)) were subcutaneously injected in the right flank of 10–12 weeks old athymic nude male mice (Charles River) suspended in 100 μL of RPMI 1640 medium or an equal volume of RPMI, along with daily intraperitoneal injections of 6-thio-dG (5 mg/kg) or vehicle once the tumor reached 100 mm^3^ (D13-D19). Mice were divided into the following experimental groups: Vehicle (H358 i.v + 5% DMSO i.p. daily between D13 and D19) and 6-thio-dG (H358 i.v. + 5 mg/kg of 6 thio-dG in 5% DMSO i.p. daily between D13 and D19). An in vivo follow-up of tumor growth was carried out. For additional details see online supplementary methods.

### Sample collection and processing

Animals corresponding to the LLC models were euthanized by intraperitoneal injection of 10 μl/g of a ketamine-xylazine anesthetic combination in saline (100:10 mg/kg respectively). Blood was collected by cardiac puncture and lungs were lavaged with 1 ml of cold PBS 1X. Right lung lobes were dissected and snap-frozen in liquid nitrogen for qPCR and ELISA analyses, and the left lung lobe was fixed in 10% buffered formalin, embedded in paraffin and cut into 3 μm sections for histopathological evaluation, immunohistochemistry (IHQ) or immunofluorescence (IFC). Animals corresponding to the human xenograft model were euthanized with the same anesthetic combination. Then, primary tumors were resected, fixed in 10% buffered formalin, embedded in paraffin and cut into 3 μm sections for IHQ and IFC. For additional details see online supplementary methods.

### Histopathological analyses and immunostaining

Hematoxylin and eosin (H&E) staining was performed for the quantification of lung tumor area and foci, and Sirius Red (Sigma-Aldrich) staining served to evaluate collagen deposition. Immunostainings were performed using the following antibodies: PanCK (Pan-Cytokeratin) (Clone AE1/AE3 1:1000, Thermo Fisher Scientific, Waltham, MA), ALU (Alu positive control probe II, Roche Diagnostics, Basel, Switzerland), HIF1A (Clone D1S7W 1:25, Cell Signaling Technology, Danvers, MA), CD31 (1:50, Abcam, Cambridge, UK), CD34 (Clone RAM34 1:100, Invitrogen, Carlsbad, CA), CD68 (Clone KP1 1:150, Santa Cruz Biotechnology), FOXP3 (Clone 221D 1:50, CNIO Monoclonal Antibodies Core Unit, Madrid, Spain), PD-1 (Clone D7D5W 1:50, Cell Signaling Technology), CD4 (Clone D7D2Z 1:50, Cell Signaling Technology), CD8 (Clone 94A 1:200, CNIO Monoclonal Antibodies Core Unit), TGFB1 (Clone V 1:100, Santa Cruz Biotechnology), E-Cadherin (Clone 36 1:1000, BD Biosciences, Franklin Lakes, New Jersey), SOX9 (1:800, EMD Millipore, Burlington, MA), Vimentin (Clone D21H3 1:50, Cell Signaling Technology), Fibronectin (1:50, Abcam), SMA (Clone 1A4 1:4, DAKO, Agilent technologies, Santa Clara, CA), H2AX (Ser139, Clone JBW301 1:200, EMD Millipore), p53 (Clone POE316A/E9 1:100, CNIO Monoclonal Antibodies Core Unit), p21 (Clone 291H/B5 1:10, CNIO Monoclonal Antibodies Core Unit), Cleaved Caspase-3 (C3, Asp175 1:300, Cell Signaling Technology) and Ki-67 (Clone D3B5 1:50, Cell Signaling Technology). Fiji open source image processing software package v1.48r (http://fiji.sc) was used for the quantification of lung tumor and collagen areas, as well as stained areas (percentage of DAB). All histological quantifications including DAB^+^ areas (%) where performed within lung tumor nodules. Quantifications were performed in 4 different fields in a random way.

### Telomere Q-FISH analyses

After deparaffinization and rehydration, tissues underwent antigen retrieval in 10 mM sodium citrate buffer and permeabilization was performed in PBS 0.5% Triton X-100 for 3 h. Next, tissues were washed 3 × 5 min in PBS 1X, fixed in 4% formaldehyde for 5 min, washed 3 × 5 min in PBS and dehydrated in a 70%–90%–100% ethanol series (5 min each) [72]. Then, the immuno-telomere-Q-FISH with the DNA damage response marker 53BP1 (1:500, Novus Biologicals, Centennial, CO) was performed and analyzed as previously described [21, 22, 73].

### Telomeric repeat amplification protocol (TRAP)

Telomerase activity was measured with a TRAP in S-100 extracts from the lung tissues as previously described [18]. Cell extracts were incubated with telomeric primers for a 60 min initial extension step at 30 °C. The extended reaction was subjected to PCR amplification (25 cycle of 30 s at 94 °C, 30 s at 59 °C, 30 s at 72 °C) in presence of 32 P end-labeled telomeric primer. The PCR reactions were resolved by 8% polyacrylamide, 7 M urea gel electrophoresis, and the gel was exposed to a phosphor-imager and scanned by a Typhoon scanner.

### ELISAS

Serum TNF and IL6 levels were quantified with TNF and IL6 Quantikine ELISA Kits (R&D systems, Minneapolis, MN). Cytokine levels were assessed in homogenized lung tissue lysates using IL10 and TGFB1 Quantikine ELISA Kits (R&D systems, Minneapolis, MN). For additional details see online supplementary methods.

### RNA isolation, reverse transcription and qPCR

Inferior right lung lobes were homogenized in TRIzol reagent (Invitrogen), and RNA was isolated using an RNeasy Mini Kit (Qiagen, Hilden, Germany) and reverse-transcribed to cDNA using SuperScript II First-Strand Synthesis System (Invitrogen). qPCR was performed as previously described [21, 22, 72, 74]. Primer sets used for qPCR are included within the supplementary information (Supplementary Table S2).

### Statistics

The number of mice used was chosen sufficiently large to reach statistical significance if so. No computational analysis was used under the experimental design. The three Rs guiding principles were applied for more ethical use of animal testing. Investigators were blinded to experimental group allocation and experiments performed. The variance between the groups that were statistically compared was similar and no data were excluded from the analysis. Following a Shapiro–Wilk normality test, either a one-way ANOVA test or a Kruskal–Wallis test were used and then, the post hoc Dunn–Sidak multiple test was carried out for multiple comparisons between experimental groups. According to the sample distribution, either a Mann–Whitney or umpaired *t* tests were used to compare differences between 2 independent groups. Survival was assessed by the Kaplan–Meier analysis, using the log Rank (Mantel–Cox) test. A Wilcoxon test was performed to assess statistical significance between *TERT* mRNA expression of tumors and adjacent tissues from LUAD and LUSC patients obtained from TIMER 2.0. For additional details see online supplementary methods.

### Reporting summary

Further information on research design is available in the Nature Research Reporting Summary linked to this article.

## Supplementary information

Supplementary information

Reporting Summary

## Data Availability

The authors declare that data supporting the findings of this study are available upon reasonable request.
