# Supplementary material for: Telomerase deficiency and dysfunctional telomeres in the lung tumor microenvironment impair tumor progression in NSCLC mouse models and patient-derived xenografts
Source: Cell Death Differ. 2023 Apr 21;30(6):1585–600. doi: 10.1038/s41418-023-01149-6 (PMC10244372; doi:10.1038/s41418-023-01149-6)
Supplement: Supplementary file 1 — Supplementary information [file 41418_2023_1149_MOESM1_ESM.docx]

***Supplementary information for***

**Telomerase deficiency and dysfunctional telomeres in the lung tumor microenvironment impair tumor progression in NSCLC mouse models and patient-derived xenografts**

**Sergio Piñeiro-Hermida^1^, Giuseppe Bosso^1,*^, Raúl Sánchez-Vázquez^1,*^, Paula Martínez^1^ and Maria A. Blasco^1,#^**

^1^Telomeres and Telomerase Group, Molecular Oncology Program, Spanish National Cancer Centre (CNIO), Melchor Fernández Almagro 3, Madrid, E-28029, Spain. These authors contributed equally: Giuseppe Bosso and Raúl Sánchez-Vázquez.

**Files included:**

**Supplementary Table S1.** Increased amplification frequency of *TERT* in NSCLC patients.

**Supplementary** **Table S2.** Primer sets used for qPCR

**Supplementary Figure S1.** Increased *TERT* mRNA expression levels in different tumor types from the TCGA.

**Supplementary Figure S2.** Increased expression of TERT in NSCLC patients is associated with a worse survival rate.

**Supplementary Figure S3.** Telomerase deficiency reduces inflammation upon lung tumor induction.

**Supplementary Figure S4.** Normal lung tissue in non LLC-challenged mice does not exhibit activation of DNA damage response, cell cycle arrest, apoptosis and proliferation.

**Supplementary Figure S5.** Telomere dysfunction mediated by 6-thio-dG diminishes inflammation upon lung tumor induction.

**Supplementary methods**

**References**

**Supplementary Table S1.** Increased amplification frequency of *TERT* in NSCLC patients.

| **Reference/source** | **Pathology** | **No. of patients** | **Amplification frequency (%)** |
| --- | --- | --- | --- |
| (1) The Cancer Genome Atlas, Firehose Legacy | Lung squamous cell carcimoma | 502 | 19.32 % |
| (2) Memorial Sloan Kettering Cancer Center, MSK-IMPACT 2021 | Lung adenocarcinoma | 186 | 18,28 % |
| (3) Cancer Genome Atlas Reseach Network, Nature 2014, 511(7511):543-550 | Lung adenocarcinoma | 230 | 17.39 % |
| (4) The Cancer Genome Atlas, Firehose Legacy | Lung adenocarcinoma | 516 | 16.86 % |
| (5) Campbell et al. Nat Genet 2016, 48(6):607-616 | Pan-lung cancer | 1144 | 14.6 % |
| (6) The Cancer Genome Atlas, PanCancer Atlas | Lung squamous cell carcimoma | 487 | 14.17 % |
| (7) Imielinski et al. Cell 2012, 150(6):1107-1020 | Lung adenocarcinoma | 183 | 12.57 % |
| (8) Chen et al. Nat Genet 2020, 52(2):177-186 | Lung adenocarcinoma | 302 | 12.25 % |
| (9) The Cancer Genome Atlas, PanCancer Atlas | Lung adenocarcinoma | 566 | 11.13 % |
| (10) Caso et al. NPJ Precision Oncology 2021, 5(1):70 | Lung adenocarcinoma | 426 | 6.34 % |
| (11) Cancer Genome Atlas Research Network, Nature 2012, 489(7417):519-525 | Lung squamous cell carcimoma | 178 | 6.18 % |
| (12) Memorial Sloan Kettering Cancer Center, MSK-IMPACT 2020 | Lung adenocarcinoma | 604 | 4.64 % |
| (13) Jordan et al. Can Discov 2017, 7(6):596-609 | Non-small cell lung cancer | 915 | 4.59 % |
| (14) Gillette et al. Cell 2020, 182(1):200-225.e35 | Lung adenocarcinoma | 110 | 3.64 % |
| (15) Rizvi et al. J Clin Oncol 2018, 36(7):633-641 | Non-small cell lung cancer | 240 | 3.33 % |

**Supplementary Table S2.** Primer sets used for qPCR.

| **Gene** | **Accession No.** | **Forward primer (5´-3´)** | **Reverse primer (5´-3´)** |
| --- | --- | --- | --- |
| ***Ccl2*** | NM_011333.3 | CACCAGCCAACTCTCACTGA | CGTTAACTGCATCTGGCTGA |
| ***Ccl12*** | NM_011331.3 | TCCTCAGGTATTGGCTGGAC | GGCTGCTTGTGATTCTCCTG |
| ***Cd163*** | NM_001170395.1 | TCTCCAGTCCAAACAACAAGC | ACCACCTCCACCTACCAAGC |
| ***Cd4*** | NM_013488.2 | ATGTGGAAGGCAGAGAAGGA | TGGGGTATCTTGAGGGTGAG |
| ***Cd8*** | NM_001081110.2 | GGAGTGGAGAAGCTAAGCCA | TGGAGCTGGAGTTCTGGAAG |
| ***Cd68*** | NM_001291058.1 | TGTTCACCTTGACCTGCTCT | TTGCAAGAGAAACATGGCCC |
| ***Egfr*** | NM_207655.2 | ACAACCCCACCACCTATCAG | GCCATCTTCTTCCACTTCGT |
| ***Foxp3*** | NM_001199347.1 | CACCCAGGAAAGACAGCAAC | CTGCACCACTTCTCTCTGGA |
| ***Hif1a*** | NM_010431.2 | TTGGAACTGGTGGAAAAACTG | ACTTGGAGGGCTTGGAGAAT |
| ***Hmox1*** | NM_010442.2 | CACGCATATACCCGCTACCT | CCAGAGTGTTCATTCGAGCA |
| ***Ifng*** | NM_008337.4 | TTCTTCAGCAACAGCAAGGC | ACTCCTTTTCCGCTTCCTGA |
| ***Il1b*** | NM_008361.3 | GCAACTGTTCCTGAACTCAACT | ATCTTTTGGGGTCCGTCAACT |
| ***Il10*** | NM_010548.2 | GCCTTATCGGAAATGATCCA | TTTTCACAGGGGAGAAATCG |
| ***Mmp9*** | NM_013599.4 | CCTGAAAACCTCCAACCTCA | GCTTCTCTCCCATCATCTGG |
| ***PD-1*** | NM_008798.2 | TCAAGGCATGGTCATTGGTA | GCTCCTCCTTCAGAGTGTCG |
| ***Rn18s*** | NR_003278.3 | ATGCTCTTAGCTGAGTGTCCCG | ATTCCTAGCTGCGGTATCCAGG |
| ***Tert*** | NM_009354.2 | TGACCAGCGTGTTAGGAAGA | CAGGAGGAAAGGAGCCAGAG |
| ***Tgfb1*** | NM_011577.2 | CGCAACAACGCCATCTATGA | ACTGCTTCCCGAATGTCTGA |
| ***Tnf*** | NM_013693.3 | GCCTCTTCTCATTCCTGCTTG | CTGATGAGAGGGAGGCCATT |

**
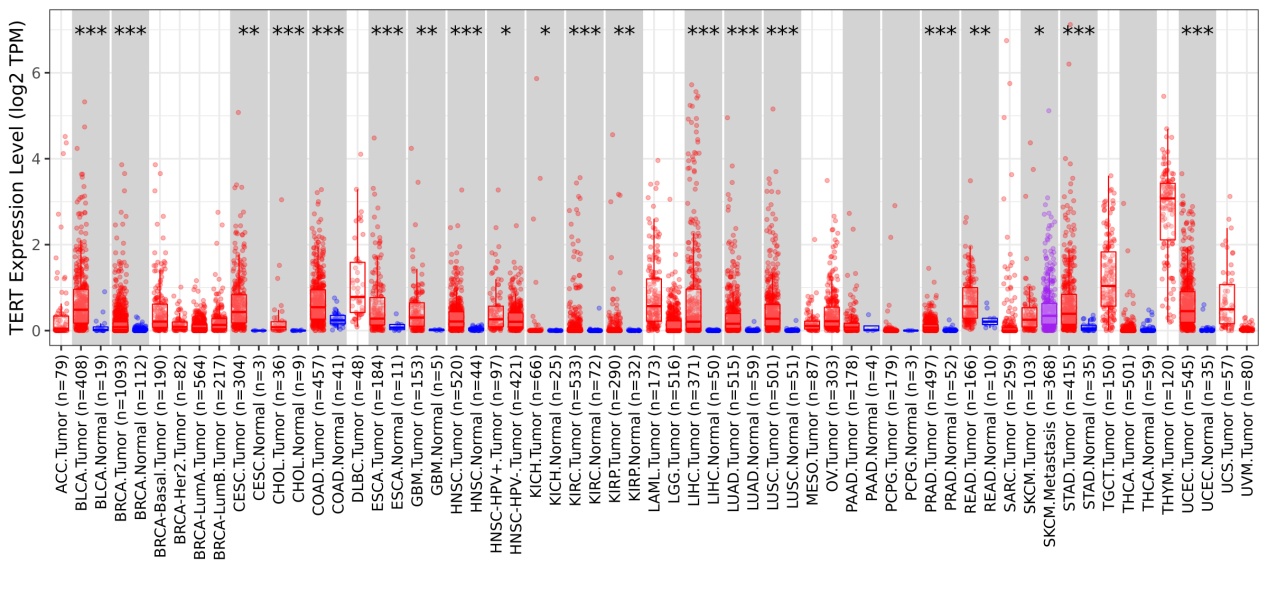
**

**Supplementary Figure S1. Increased *TERT* mRNA expression levels in different tumor types from The Cancer Genome Atlas (TCGA).** TERT mRNA expression data in different tumor types and adjacent normal tissues obtained from the TIMER 2.0 database. Data are expressed as mean ± SEM (the number of patients is indicated in each case). *p<0.05; **p<0.01; ***p<0.001 (Wilcoxon test). LUAD (lung adenocarcinoma) and LUSC (lung squamous cell carcinoma).

**
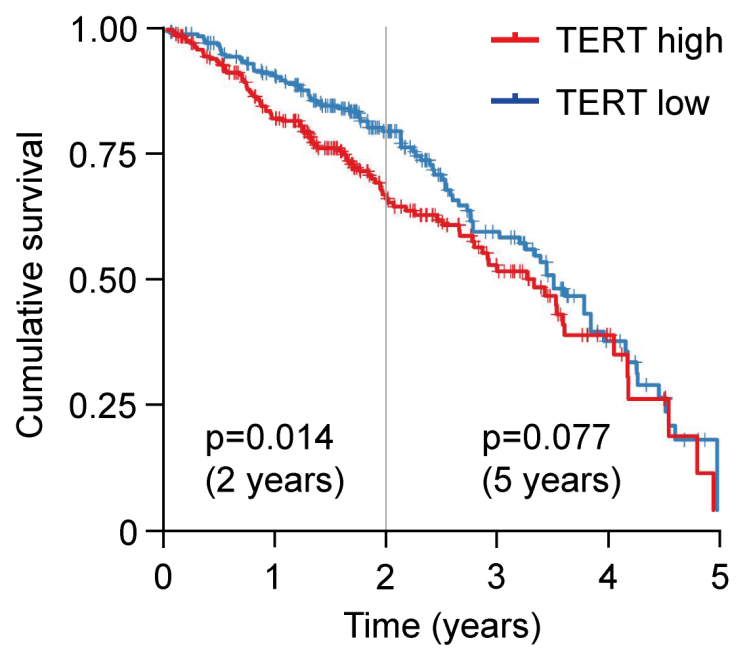
**

**Supplementary Figure S2.** **Increased expression of TERT in NSCLC patients is associated with a worse survival rate.** Cumulative survival in NSCLC patients with high and low TERT expression obtained from the TIMER 2.0 database (data from The Cancer Genome Atlas (TCGA)).

**
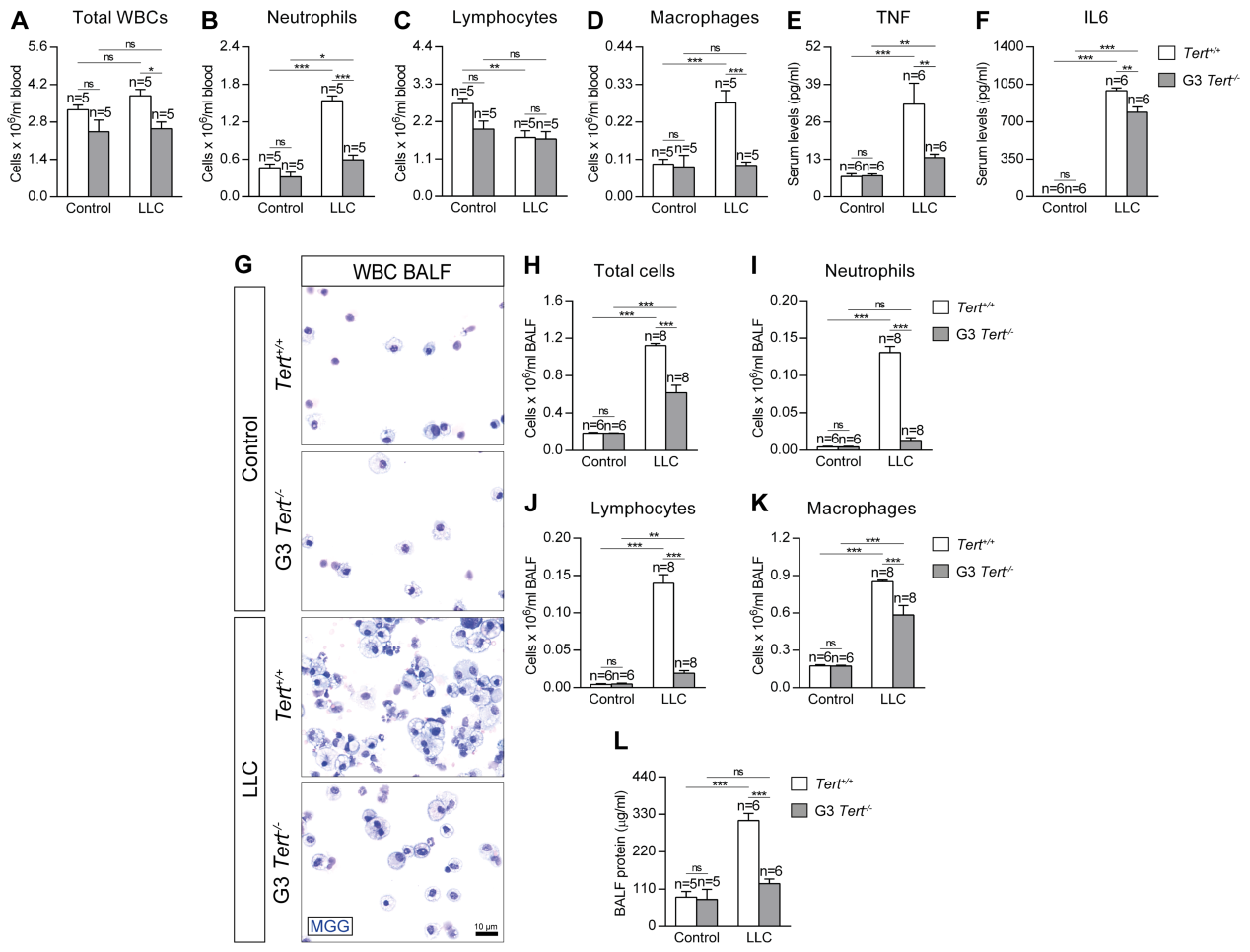
**

**Supplementary Figure S3. Telomerase deficiency reduces inflammation upon lung tumor induction.** (A-F) Quantification of total white blood cells (A), neutrophils (B), lymphocytes (C) and macrophages (D) in peripheral blood, and TNF (E) and IL6 (F) protein levels in serum from LLC-challenged *Tert^+/+^* and G3 *Tert^-/-^* mice and controls. (G-L) Representative BALF cytospin preparations (May-Grünwald Giemsa (MGG)) (G), and quantification of total (H) and differential BALF cell counts for neutrophils (I), lymphocytes (J) and macrophages (K), and total protein concentration in BALF (L) of *Tert^+/+^* and G3 *Tert^-/-^* mice.

**
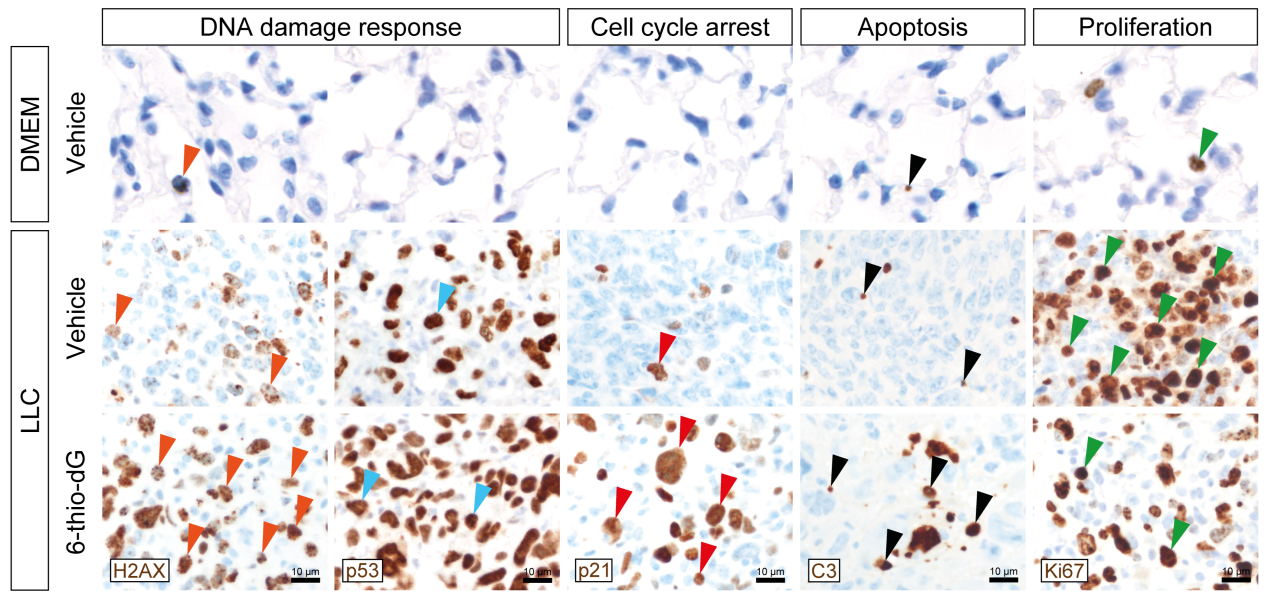
**

**Supplementary Figure S4.** **Normal lung tissue in non LLC-challenged mice does not exhibit activation of DNA damage response, cell cycle arrest, apoptosis and proliferation.** Representative lung immunostainings for γH2AX (brown; orange arrowheads H2AX^+^ cells), p53 (brown, blue arrowheads indicate p53^+^ cells), p21 (brown; red arrowheads indicate p21^+^ cells), Cleaved Caspase-3 (C3, brown; black arrowheads indicate C3^+^ cells), and Ki67 (brown; green arrowheads indicate Ki67^+^ cells) in lung sections from non-LLC challenged mice (DMEM + vehicle) and LLC-challenged mice (LLC + vehicle and LLC + 6-thio-dG).

**
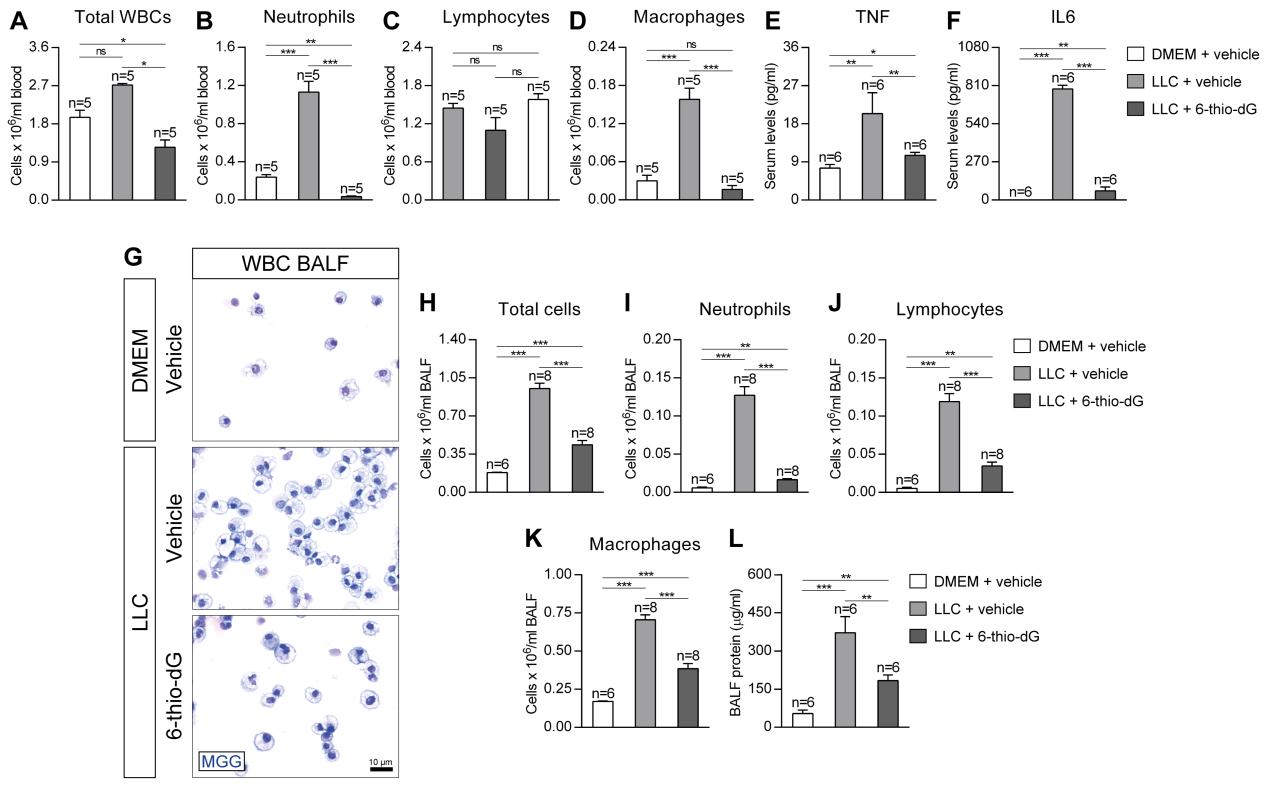
**

**Supplementary Figure S5. Telomere dysfunction mediated by 6-thio-dG diminishes inflammation upon lung tumor induction.** (A-F) Quantification of total white blood cells (A), neutrophils (B), lymphocytes (C) and macrophages (D) in peripheral blood, and TNF (E) and IL6 (F) protein levels in serum from LLC-challenged mice treated with 6-thio-dG vs. controls. (G-L) Representative BALF cytospin preparations (May-Grünwald Giemsa (MGG)) (G), and quantification of total (H) and differential BALF cell counts for neutrophils (I), lymphocytes (J) and macrophages (K), and total protein concentration in BALF (L) of LLC-challenged mice treated with 6-thio-dG vs. controls.

**Supplementary methods**

**Data from NSCLC patients**

mRNA expression was assessed using an Illumina HiSeq sequencing system, as indicated in the cBioPortal website. Copy number variation (CNV) data were obtained as previously described (1). Genomic libraries were obtained upon extraction, purification and digestion of genomic DNA from tissue samples of NSCLC patients. Such DNA was further hybridized onto Affymetrix SNP6.0 arrays and normalized as previously described (1). Recurrent peaks for focal somatic copy number alteration were identified using GISTIC 2.0. A peak was considered focally amplified within a tumor if the GISTIC 2.0-estimated focal copy number ratio was greater than 0.1 (1).

**Lewis lung carcinoma (LLC) models**

*Tert^+/-^* mice were intercrossed to generate *Tert^+/+^* and first generation (G1) homozygous Tert^-/-^ mice. Second generation (G2) *Tert^-/-^* mice were generated by successive breeding of G1 *Tert^-/-^* and then third generation (G3) *Tert^-/-^* mice by crosses between G2 *Tert^-/-^* mice (Figure 1E). The generation of the Lewis lung carcinoma (LLC) models was performed using the cell line LL/2 (LLC1) (ATCC® CRL-1642™) obtained from the American Type culture collection (ATCC). This cell line was maintained in Dulbecco's Modified Eagle's Medium (DMEM), supplemented with 10% fetal bovine serum and an antibiotic cocktail containing penicillin-streptomycin.

**Human NSCLC xenograft model**

The generation of the human NSCLC xenograft model was performed using the H358 cell line (human bronchoalveolar carnicoma; NCI-H358 [H-358, H358] (ATCC® CRL-5807™) obtained from the ATCC. This cell line was maintained in RPMI 1640 medium, supplemented with 10% fetal bovine serum and an antibiotic cocktail containing penicillin-streptomycin. The tumor volume was assessed using a precision calliper and employing the formula volume = (l+w^2^)/2; l: length; w: width, using the largest axes of the tumor).

**Sample collection and processing**

Serum was obtained by centrifugation at 3000 xg for 10 min at 4 °C and stored at -80°C until further. On the other hand, bronchoalveolar lavage fluid (BALF) was centrifuged at 10000 rpm for 5 min at 4 °C and the supernatants were stored at -80 °C to subsequently assess total protein concentration in BALF using the Pierce BCA Protein Assay Kit (Thermo Fisher Scientific, Waltham, MA, USA). Hereafter, the BALF pellets were resuspended in 500 μl of ACK Lysing Buffer (Thermo Fisher Scientific) and centrifuged at 10000 rpm for 5 min at 4 °C after 10 min of incubation. The supernatants were discarded and 500 µl of PBS 1X were added to the pellet to prepare the BALF cytospin preparations by centrifugation of the slides at 1500 rpm for 5 min.

**Quantification of BALF**

Total cell number was counted and expressed as cells/ml of BALF, and differential cell counts were performed on May-Grünwald Giemsa (Sigma-Aldrich)-stained cytospins, counting a minimum of 300 cells per slide. Determination of differential cell counts was performed using standard morphology criteria.

**ELISAS**

Superior right lung lobes were homogenized in RIPA Buffer (Thermo Fisher Scientific) containing a protease-phosphatase inhibitor mixture (Roche, Basel, Switzerland).

**Statistics**

Statistical analyses were accomplished using SPSS Statistics Software v21 for Windows (IBM, Armonk, NY, USA). For all analyses, a p value<0.05 was considered statistically significant. Results are shown as mean values ± standard error of the mean (SEM). For all analyses, a P value < 0.05 was considered statistically significant.

**References**

1. Campbell JD, Alexandrov A, Kim J, Wala J, Berger AH, Pedamallu CS, et al. Distinct patterns of somatic genome alterations in lung adenocarcinomas and squamous cell carcinomas. Nat Genet. 2016;48:607–16.
